# The Deleted in Brachydactyly B Domain of ROR2 Is Required for Receptor Activation by Recruitment of Src

**DOI:** 10.1371/journal.pone.0001873

**Published:** 2008-03-26

**Authors:** Shiva Akbarzadeh, Lee M. Wheldon, Steve M. M. Sweet, Sonia Talma, Faraz Khosravi Mardakheh, John K. Heath

**Affiliations:** CR-UK Growth Factor Group, School of Biosciences, University of Birmingham, Edgbaston, Birmingham, United Kingdom; University of California, Berkeley, United States of America

## Abstract

The transmembrane receptor ‘ROR2’ resembles members of the receptor tyrosine kinase family of signalling receptors in sequence but its' signal transduction mechanisms remain enigmatic. This problem has particular importance because mutations in ROR2 are associated with two human skeletal dysmorphology syndromes, recessive Robinow Syndrome (RS) and dominant acting Brachydactyly type B (BDB). Here we show, using a constitutive dimerisation approach, that ROR2 exhibits dimerisation-induced tyrosine kinase activity and the ROR2 C-terminal domain, which is deleted in BDB, is required for recruitment and activation of the non-receptor tyrosine kinase Src. Native ROR2 phosphorylation is induced by the ligand Wnt5a and is blocked by pharmacological inhibition of Src kinase activity. Eight sites of Src-mediated ROR2 phosphorylation have been identified by mass spectrometry. Activation via tyrosine phosphorylation of ROR2 receptor leads to its internalisation into Rab5 positive endosomes. These findings show that BDB mutant receptors are defective in kinase activation as a result of failure to recruit Src.

## Introduction

Human ROR2 is one of two related ROR proteins, identified by sequence similarity to TRK receptors [1], whose mechanism of action remains enigmatic. From structural predictions ROR receptors contain an extra cellular immunoglobulin-like domain; a cysteine-rich domain (CRD) that resembles the Wnt binding domain of Frizzled (Fz) receptors; a Kringle motif and single pass transmembrane domain. The cytoplasmic region contains a putative kinase domain and a bipartite C-terminal serine/threonine-rich and proline-rich region [1] containing potential effector protein docking sites.

Mutations in *ROR2* cause two skeletal disorders in humans, autosomal recessive Robinow Syndrome (RS) and autosomal dominant Brachydactyly type B (BDB) [2]-[5]. RS is characterised by short-limbed dwarfism and costovertebral defects. By contrast BDB is characterised by shortening of digits, often missing nails and phalangeal bones, but otherwise normal phenotype. *ROR2* mutations causing RS frequently result in truncation of the receptor in either extra cellular or cytoplasmic regions and are predicted to be loss of function. ROR2-deficient mice exhibited severe skeletal defects which are analogous to those of human RS mutations including dwarfism as well as heart and lung malformation [6], [7]. In contrast, *ROR2* mutations causing BDB result in truncation of the cytoplasmic region, either immediately before or after the kinase domain and, by virtue of their dominant acting functions, are predicted to be associated with gain of function or dominant negative activity.

Despite the significant role ROR2 plays in mammalian skeletogenesis the molecular mechanism by which it exerts its biological effects remain elusive. This study aimed to investigate the role of kinase activation in ROR2 signalling and to determine the role of the C terminal domain deleted in BDB.

A growing body of evidence implicates members of the Wnt family of signalling molecules as endogenous ROR2 effectors. Functional studies in developing xenopus embryos have demonstrated the role of ROR2 in non-canonical Wnt pathways [8], [9]. In mammals, ROR2 has been shown to bind to a number of canonical and non-canonical Wnts and mediate non-canonical Wnt signalling in cultured cells [10]–[13]. We find that a constitutively dimerised form of ROR2 exhibits kinase activity and that Wnt5a-induces activation of Wt ROR2 kinase activity and internalisation of ROR2 into Rab5 endosomes. We also show that activation of ROR2 kinase requires the C terminal domain that is deleted in BDB and that this domain is also required for recruitment of the non receptor kinase Src. Native ROR2 is a target for Src mediated phosphorylation and pharmacological inhibition of Src suppresses ROR2 activation. Collectively these findings reveal that the BDB mutant ROR2 receptors are defective in kinase activation via failure to recruit Src.

## Results and Discussion

### ROR2 Exhibits Intrinsic Kinase Activity

**Figure 1:**
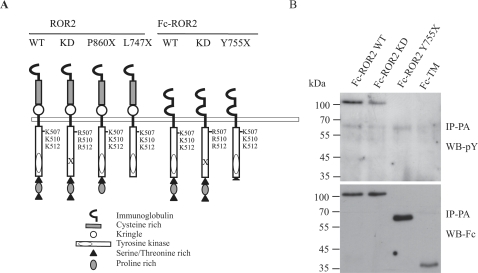
Fc-ROR2 WT is tyrosine phosphorylated. A) A schematic diagram of the full length ROR2 and Fc-ROR2 chimeras used in this study. ‘Kinase dead’ (KD) mutants were constructed by mutating three Lysine residues within the ATP binding pocket to Argenine. B) Fc fusion chimeras of the intracellular domains from ROR2 WT, ROR2 KD, ROR2 Y^755^X and, as a control, the Fc-transmembrane domain of FGFR1 fusion were expressed in chondrocytes. The chimeric receptors were immunoprecipitated using Protein-A-sepharose (IP-PA) and analysed by Western blotting following 10% SDS-PAGE. Receptor phosphorylation was detected by a cocktail of phosphotyrosine antibodies (4G10/pY20; WB-pY, upper panel). The membrane was stripped and reprobed with anti-IgG1 antibody to compare receptor expression levels (WB-Fc, lower panel).

To evaluate the intrinsic kinase activity potential of ROR2, the cytoplasmic domain of ROR2 was fused to the dimeric Fc portion of human IgG to create Fc-ROR2 ‘wild type’ (WT), Fc-ROR2 ‘Kinase dead’ and an Fc-ROR2 truncation mutant (Y^755^X) [14]. Fig. 1A shows a schematic detailing these constructs as well as the original full length, myc-tagged ROR2 constructs. Following expression in T/C28a2 human chondrocytes, robust Fc-ROR2 WT phosphorylation was detected in contrast with a markedly reduced phosphorylation of Fc-ROR2 KD. This suggests that, despite divergence from the RTK consensus sequence [15], ROR2 has intrinsic kinase activity which is elicited upon dimerisation (Fig. 1B, upper panel). This confirmed the recent reports that ROR2 is phosphorylated upon homo-dimerisation [16]. It was noted in these experiments that, although markedly reduced compared to WT, residual receptor phosphorylation was observed in cells expressing Fc-ROR2 KD, raising the possibility there may be other tyrosine kinases or co-receptors involved in ROR2 receptor phosphorylation, although the presence of residual endogenous kinase activity in the mutant cannot be rigorously excluded.

A truncated receptor variant, Fc-ROR2 Y^755^X lacking the C-terminal region of ROR2 and emulating the BDB mutant form of ROR2, was also expressed in chondrocytes and evaluated for tyrosine phosphorylation. Fc-ROR2 Y^755^X exhibited markedly reduced levels of tyrosine phosphorylation compared to Fc-ROR2 WT (despite equivalent levels of expression, Fig. 1B, lower panel) resembling that observed by mutation of the ATP binding pocket in Fc-ROR2 KD. This suggests either the majority of tyrosine residues that are phosphorylated in the Fc-ROR2 WT protein are located C-terminal to Y^755^ or the reduced phosphorylation observed in Fc-ROR2 Y^755^X is due to lack of binding sites for non-receptor tyrosine kinases that may contribute to ROR2 phosphorylation. In either case, this result highlights the essential role of ROR2 C-terminal for its full activation.

### Wnt5a activates Tyrosine Phosphorylation of ROR2

**Figure 2:**
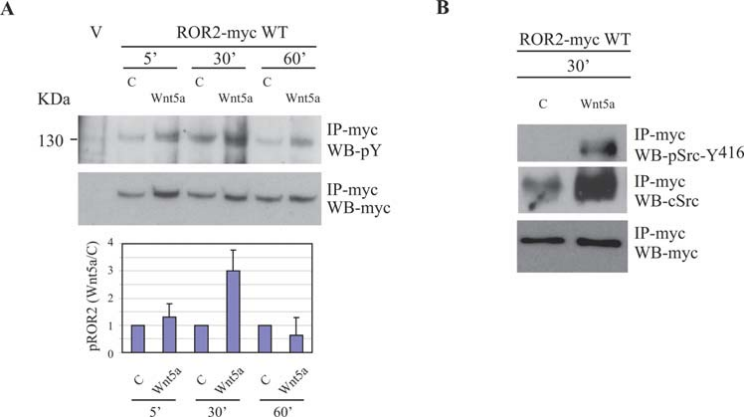
ROR2 receptor exhibits increased tyrosine phosphorylation and Src association upon Wnt5a stimulation. A) Chondrocytes were either transfected with pcDNA3.1 (V, control) or pcDNA3.1 containing the myc-tagged, wild type, ROR2 construct (ROR2-myc WT). Following 1 hr of serum starvation in KHB, cells were either stimulated with 0.1% BSA carrier (C) or Wnt5a (1.6 µg/ml) for 5, 30 or 60 min. Control vector-transfected cells (V) were not stimulated. The receptor was immunoprecipitated using anti-myc antibody (IP-myc) and analysed by SDS-PAGE and subsequent Western blotting. The membrane was probed with an anti-phosphotyrosine cocktail (WB-pY), followed by stripping and re-probing with anti-myc antibody (WB-myc). The fold-increase of ROR2 receptor phosphorylation was determined by normalizing Wnt5a-induced phosphorylation to carrier-induced phosphorylation (mean±s.e.m., n = 3, lower panel). B) Chondrocytes expressing ROR2-myc WT were stimulated with either 0.1% BSA (C) or Wnt5a (1.6 µg/ml) for 30 min. The receptor was immunoprecipitated using anti-myc antibody (IP-myc) and probed with a phospho-Src antibody (WB-pSrc-Y^416^) to detect activated Src. The membrane was stripped and reprobed with anti-c-Src (WB-cSrc) and anti-myc (WB-myc).

As discussed above, members of the Wnt family of ligands have been implicated in ROR2 signalling. Having established that ROR2 could exhibit intrinsic kinase activity (albeit of an unusual kind) we were interested in discovering if Wnt ligands could elicit activation of the kinase activity of native ROR2 receptors. Chondrocytes transfected with full length myc-tagged ROR2 (ROR2-myc WT) were stimulated with either BSA carrier or Wnt5a for 5, 30 or 60 min. The receptor was immunoprecipitated and receptor phosphorylation was detected by phosphotyrosine immunoblotting (Fig. 2A, upper panel). A marked increase in receptor tyrosine phosphorylation after 30 min of Wnt5a stimulation was detected compared to carrier stimulated controls. These findings show that Wnt5a ligand elicits tyrosine phosphorylation of native ROR2, confirming that Wnt5a is, directly or indirectly, a bona fide activator of ROR2 signalling, acting via stimulation of intrinsic tyrosine kinase activity.

### Src Phosphorylates ROR2

The foregoing data are consistent with Wnt5a-mediated dimerisation of ROR2 being a primary mechanism of receptor activation but do not eliminate the engagement of non-receptor tyrosine kinases or co-receptors in this process. This possibility is strengthened by the presence of residual C-terminal phosphorylation in the Fc-ROR2 KD construct. We accordingly sought candidate non-receptor tyrosine kinases that could employ dimerised ROR2 as a substrate.

A notable feature of the anti-phosphotyrosine blots of immunoprecipitated native ROR2 stimulated with Wnt5a was a tyrosine phosphorylated protein of ∼60 KDa (data not shown). Src family kinases have been directly implicated in signalling from a number of RTKs [17] and therefore the possibility that this unknown protein was a Src family kinase was further investigated. Immunoprecipitated ROR2 was probed for active Src (using anti-phospho-Src Y^416^) and an immunoreactive protein, at the predicted M_W_ of Src, was recruited to the receptor upon 30 min of Wnt 5a stimulation (Fig. 2B, upper panel). This result indicates that recruitment and activation of Src kinase is a consequence of Wnt5a stimulation of ROR2. It should be noted that the C-terminal region of ROR2, deleted in BDB, contains both putative consensus Src SH2 domain (Y^786^XXP, Y^873^VTT) [18] and SH3 (R^809^PMVPPP) domain [19] recognition sites.

**Figure 3:**
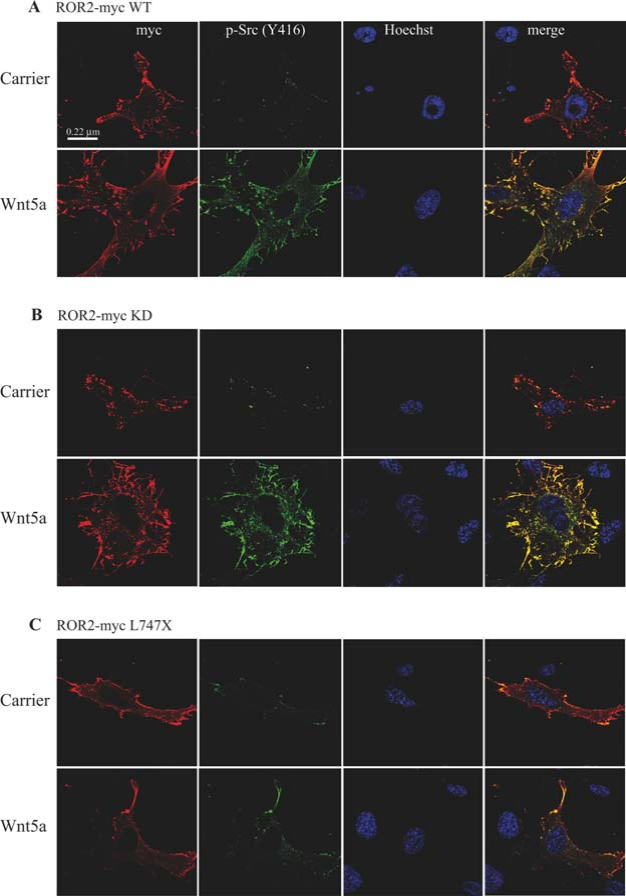
Wnt5a-induced association of activated Src and the ROR2 receptor. Chondrocytes were transfected with; A) ROR2-myc WT, B) ROR2-myc KD or C) ROR2-myc L^747^X and serum-starved for 1 hr in KHB prior to stimulation with either 0.1% BSA carrier or Wnt5a (1 µg/ml) plus heparin (10 µg/ml) for 30 min. The cells were fixed and stained with anti-myc, anti-phospho-Src (Y^416^) and Hoechst.

Chondrocytes expressing ROR2-myc constructs exhibited slightly elevated Src activity when compared to non-ROR2 expressing cells and Src activity was dramatically increased in ROR2 expressing cells upon Wnt5a stimulation (Fig. 3). Active Src co-localised with ROR2-myc WT in both cell surface and intracellular locations indicative of a role in intracellular trafficking (Fig. 3A). We also noted in these experiments that ROR2-myc WT transfected cells exhibited extensive filopodia as reported previously [12]. ROR2-myc KD, similar to WT, co-localised with active Src (Fig. 3B) contrasting with the expression of ROR2-myc L^747^X, which not only resulted in lower Src activation even after Wnt5a stimulation, compared to either ROR2-myc WT or KD, but also exhibited far less co-localisation (Fig. 3C).

**Figure 4:**
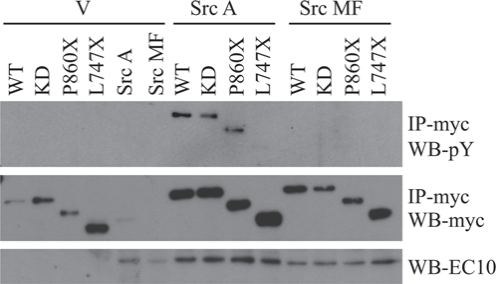
ROR2 receptor is a substrate for Src. Chondrocytes were co-transfected with myc-tagged ROR2 WT, ROR2 KD and myc-tagged deletion mutants ROR2 P^860^X and ROR2 L^747^X and either vector alone (V), constitutively active Src (Src A) or inactive Src (Src MF). Lanes 5 and 6 are controls for Src A and Src MF, respectively. The receptor constructs were immunoprecipitated with anti-myc (IP-myc) and probed with anti-phosphotyrosine antibodies (WB-pY, upper panel) then stripped and reprobed with anti-myc (loading control, WB-myc). Src A and MF expression was confirmed on whole cell lysate prior to immunoprecipitation using anti-chicken Src antibody (WB-EC10, lower panel).

We next sought to identify the ROR2 cytoplasmic domain(s) that are essential for Src kinase binding. Full length ROR2-myc WT, KD, P^860^X and L^747^X were co-expressed in chondrocytes with either a constitutively active Src Y^527^F (Src A) or a kinase dead Src K^295^M/Y^527^F (Src MF). ROR2-myc WT, KD, and P^860^X receptors were tyrosine phosphorylated in a ligand-independent manner when co-expressed with Src Y^527^F (Fig. 4). Phosphorylation of L^747^X mutant was almost ablated compared to the WT receptor, indicating that the C-terminal proline-rich and/or the first serine/threonine-rich regions are essential for Src binding. No receptor phosphorylation was observed when Src MF was co-expressed with any of the ROR2-myc constructs.

Collectively, results in figures 2 and 3 establish that Wnt5a activation of ROR2 results in Src kinase activation and that activated Src kinase is able to phosphorylate ROR2 upon tyrosine residues. Thus Wnt5a stimulation results in two pathways of ROR2 phosphorylation: one via receptor dimerisation and a second via recruitment of Src. The consequence of these combined actions is prominent phosphorylation of the receptor on tyrosine residues.

We next sought to identify the substrates for Src-mediated phosphorylation in ROR2. Full length ROR2-myc WT was co-expressed with Src A, immunoprecipitated and tryptic peptides subjected to CID MS/MS (Supplementary Fig. S1 and Supplementary Tables S1 and S2). Eight Src-dependent ROR2 phosphorylation sites were identified by mass spectrometry with 48.6% sequence coverage including the dual tyrosines Y^645^/Y^646^ in the predicted activation loop of ROR2 and Y^873^ in the BDB domain.

### Wnt5a induces ROR2 internalisation into Rab5 positive vesicles

**Figure 5:**
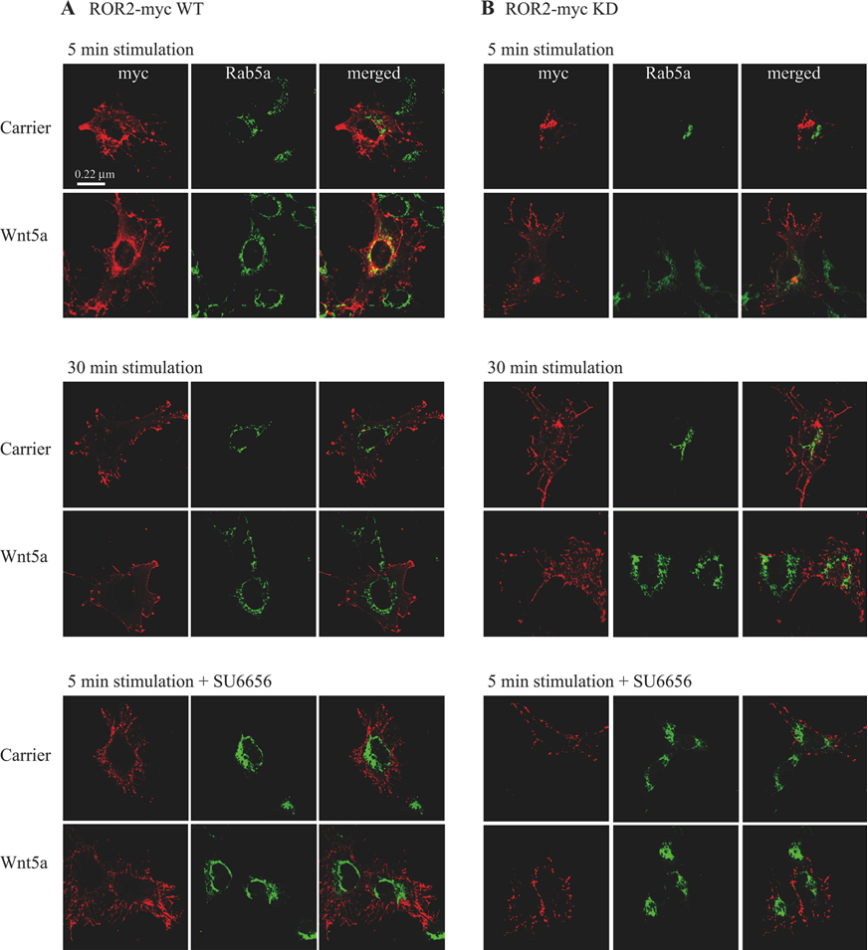
ROR2 is targeted into Rab5A-positive endosomes upon Wnt5a stimulation. A) ROR2-myc WT and B) ROR2-myc KD transfected chondrocytes were serum-starved in KHB for1 hr. Cells were stimulated with either 0.1% BSA carrier or Wnt5a (1 ug/ml) plus heparin (10 µg/ml) for 5 min (upper panels), 30 min (middle panels) or 5 min in the presence of 24 µM SU6656 (lower panels). The cells were fixed and subsequently stained with anti-myc and anti-Rab5A antibodies.

Activation of RTK kinase activity results in internalisation of activated receptor complexes resulting in both signal propagation and degradation. Receptors internalised via kinase-dependent pathways are initially targeted into early endosomes before sorting for either recycling or degradation [20]. The vesicular sorting Rab 5 GTPase is localised to early endosomes, regulates plasma membrane-endosome fusion [21] and, at least for some RTKs [22] influences signalling outcomes. If Wnt5a activation of ROR2 phosphorylation yields a productive signalling response it is predicted that internalisation of ROR2 into Rab5 positive vesicles would occur. ROR2-myc WT or KD transfected cells were stimulated with either 0.1% BSA carrier or Wnt5a and the sub-cellular localisation of ROR2 and Rab5A were examined by immunofluorescence. Clearly ROR2-myc WT co-localisation with Rab5A was increased after 5 min but not after 30 min (Fig. 5A, upper and middle panels, respectively) of Wnt5a stimulation. These findings show that activation of ROR2 phosphorylation via Wnt5a results in rapid receptor internalisation into Rab5 positive sorting endosomes. It also provides a functional readout for ROR2 signalling although, given ignorance of the downstream signalling pathways employed by ROR2, this could indicate either attenuation or sustained signalling. Given the prominent role for Src in ROR2 kinase activation described above we evaluated the role of Src kinase activity in the Wnt-induced localisation into Rab5 endosomes. ROR2-myc WT transfected chondrocytes were incubated with a Src family kinase inhibitor (SU6656) prior to stimulation with 0.1% BSA carrier or Wnt5a for 5 min (Fig. 5A, lower panel) and the effect on sub-cellular localisation of ROR2 was detected by immunofluorescence. ROR2 showed little co-localisation with Rab5 when Src was pharmacologically inhibited. Collectively, these results show that Src activity is a requirement for ROR2 trafficking into the Rab5 positive endosomal compartment following activation by Wnt5a. Interestingly, despite the ‘latent’ phosphorylation of Fc-ROR2 KD (Fig. 1B), only marginal co-localisation with Rab5A was observed after 5 min of Wnt5a stimulation (Fig. 5B, upper panel) and no co-localisation after either 30 min or 5 min following treatment with SU6656 (Fig. 5B, middle and lower panels, respectively). No co-localisation was ever observed when cells were stimulated with 0.1% BSA carrier.

This leads to an “ignition trigger” model of ROR2 activation whereby low level intrinsic ROR2 kinase activity is sufficient to recruit and activate Src which amplifies ROR2 receptor phosphorylation to the full active state. This jointly explains the apparently weak kinase activity, the requirement for an intact ATP binding site, the requirement of functional Src kinase and the key role of the BDB domain in ROR2 function. Our findings indicate that ROR2 represents a second non-canonical pathway for Wnt signalling which is an unorthodox example of classical RTK mediated signalling which proceeds by ligand-mediated receptor homo-dimerisation followed by tyrosine phosphorylation of receptor substrate residues and propagation of the signal. In support of this model we show that dimerisation is sufficient to induce ROR2 activation and that Wnt5a induces ROR2 phosphorylation. A secondary role for co-receptors in ROR2 signalling [13], [23] cannot however be ruled out on the basis of the evidence presented here but these would of necessity involve pathways that do not involve either tyrosine kinase activity or BDB domain of ROR2.

### Biological Implications

We now turn to the significance of these findings for understanding the pathology of human BDB mutations in ROR2. Our results show that the region deleted in BDB mutants is required for efficient ROR2 phosphorylation. This region also contains potential binding sites for Src and target sites for phosphorylation and recruitment of potential downstream effectors. Thus deletion of this region, as found in BDB mutants, yields a receptor which is competent to bind Wnt ligand but unable to produce a productive tyrosine phosphorylation signalling response. In classical RTK signalling receptors this type of mutant would, depending upon gene dosage, be predicted to exhibit dominant negative properties which diminish the signalling functions of endogenous native receptors in the presence of ligand. This would suggest that heterozygous BDB mutations (if expressed at appropriate levels) would resemble loss of function homozygous RS mutants. This is clearly not the case as the phenotypes of the two syndromes are quite distinct. The alternative explanation is that BDB mutations represent a gain of function phenotype whereby ablation of the ROR2 tyrosine kinase pathway results in the activation (or relief of repression) of signalling pathways in the limb which leads to digit truncation [24]. Candidate pathways would include those repressed by Src kinase activity and limited to digit primordia and not other regions of ROR2 expression [24].

## Methods

### Cell Culture and Transfection

T/C-28a2 human chondrocytes (a generous gift of Dr. Goldring, Saint Louis University, USA) were cultured at 37°C, 5% CO_2_ for up to six months in a mixture of Dulbecco's modified Eagle medium and Ham's F12 (1∶1, v/v) supplemented with 2 mM glutamine (Invitrogen), 0.1 mg/mL streptomycin, 0.2 U/mL penicillin, 4.5 g/L glucose (Sigma) and 10% fetal calf serum (v/v) (Labtech International). Cells were transfected with Lipofectamine 2000 (Invitrogen) according to manufacturer's instruction except using only half the amount of plasmid DNA and Lipofectamine 2000 reagent recommended.

### Reagents and Antibodies

Recombinant murine Wnt5a was obtained from R&D Systems. SU6656 Src inhibitor (used at 24 µM) was purchased from CALBIOCHEM. Primary antibodies used in this study were: mouse anti-IgG-Fc-HRP conjugate and mouse anti-IgG (Pierce); mouse anti-myc; rabbit anti-phospho-Src Y_416_ (Cell Signalling); the anti-phosphotyrosine cocktail used in western blotting comprised mouse anti-phospho-tyrosine clone 4G10™ (Upstate) and clone pY20 (ICN Biomedicals, Inc.); rabbit anti-Rab5A and rabbit anti-cSrc (Santa Cruz). The secondary antibodies were anti-mouse- and anti-rabbit-IgG Horseradish Peroxidase conjugates (Amersham). The secondary antibodies for immunofluorescence studies were anti-mouse-Alexa 546 (Molecular Probe) and anti-rabbit-FITC (DAKO).

### cDNA Constructs

The pcDNA3.1 mouse myc-tagged ROR2 was a generous gift of Dr. Minami (Kobe University, Japan). The chicken constitutively active Src (Src Y^527^F, Src A) and kinase dead Src (Src Y^527^F, K^295^M, Src MF) in pBabe Puro were generous gifts of Dr. Frame (The Beatson Institute for Cancer Research, UK).

ROR2 L^747^X was amplified using a primer pair that introduced a *Xho*I restriction site at the point of truncation: XF (5′-AAGGAAAAAAGCGGCCCTCGAGGTCGACCCACGCGTCCG-3′) and L^747^XR (5′-ATAATCCTCGAGGAGCCGGCTGTGGATGTC-3′). The amplified product was digested with *Xho*I restriction enzyme and sub-cloned into pcDNA3.1-myc. Similarly, the ROR2 P^860^X mutant was constructed using XF and P^860^XR (5′-CAGTTCCTCGAGCGGCTTGGGGACCATCTG-3′) primers.

The Fc-ROR2 WT in pEFBOS was constructed by inserting the transmembrane, cytoplasmic and the 3′ flanking region of ROR2 (IMAGE clone 2231989; a generous gift of Dr. Wilkie, Oxford University, UK), into ss-Fc-IRES-Tpz-pEFBOS vector. The Fc-ROR2 Y^755^X truncation mutant was generated by overlap extension mutagenesis. Two PCR reactions using two mutagenic primers 6Lstop (5′-CTTTCCAATTAAAACAGCTCGG-3′) and 6Rstop (5′-CCGAGCTGTTTTAATTGGAAAG-3′) that introduced a stop codon at Y^755^ and a silent *Mse*1 restriction site for selection and two outside primers (5R: 5′-GATCTGCACCGGGTAGAAGTTG-3′ & 5L: 5′-CAACCAGGATGTGGTGGAGATG-3′). The kinase dead mutations (K^507,510,512^R) of both full length and Fc-conjugated ROR2 were generated using QuickChange® II XL Site-Directed Mutagenesis Kit (Stratagene) according to manufacturer instructions using primers 3K-3RF (5′-GGCCGTGGCCATCAGGACGCTGAGAGACAGGGCTGAGGGGCCCC-3′), and the second primer that was the reverse complementary sequence to 3K-3RF.

### Wnt5a stimulation, Immunoprecipitation and Western Blotting

Following serum starvation in Krebs Hepes Buffer (KHB) chondrocytes were stimulated with either carrier control (0.1% BSA in PBS (w/v)) or Wnt5a (1.0-1.6 µg/mL). The reaction was stopped by adding 10x volume cold PBS. Cells were lysed in lysis buffer (150 mM NaCl; 10 mM Tris-HCl; 1 mM EGTA; 1 mM EDTA; 1% NP-40 (v/v); 1 mM Na_3_VO_4_; 50 mM NaF and 1 tablet of complete protease inhibitor cocktail (Roche) per 10 ml of buffer, PH 7.5). For ROR2-myc immunoprecipitation, 200–500 µg whole cell lysate was incubated with 0.5 µg anti-myc antibody for 1 hr at 4°C followed by incubating the mixture with 30 µl of a 50% protein A-sepharose slurry (Amersham Biosciences) for 1 hr hour at 4°C. The immunoprecipitates were washed excessively and boiled in 2 × SDS sample buffer (100 mM Tris-HCl; 10% glycerol (v/v); 2% SDS (w/v); 0.1% bromophenol blue (w/v); 200 mM DTT; pH 6.8) for 5 min at 95°C prior to running on SDS PAGE.

Gels were transferred to PVDF membrane (Millipore) and blocked in TBS-T containing 5% bovine serum albumin (BSA, w/v). Primary antibodies (in TBS-T/5% BSA) were incubated with the membrane for 1 hr at room temperature or 4°C overnight. The membrane was washed (3×15 min) in TBS-T and subsequently probed with the conjugated secondary antibody (in TBS-T/1% BSA) for 45 min at room temperature. The membrane was washed (5×10 min) with TBS-T, before membranes were exposed to ECL reagents (Pierce) for visualization of immunoreactive proteins.

### Immunofluorescence

T/C-28a2 chondrocytes were grown on acid scratched glass cover slips. Following Wnt5a stimulation, cells were washed in cold PBS and fixed in 4% paraformaldehyde (w/v) for 10 min at room temperature. Cells were then washed 3 times in PBS and permeabilised in 0.2% triton X-100 (v/v)/ 0.5% BSA (w/v) for 3-5 min, followed by washing with PBS. Non-specific binding sites were then blocked with 4% BSA/TBST (w/v) for 10 min. The cover slips were incubated in a cocktail of primary antibodies (1: 100 in 4% BSA/TBST (w/v)) for one hour, followed by incubation in each secondary antibody (1: 100 in 4% BSA/TBST (w/v)) separately with PBS washes between each staining. The cover slips were mounted onto glass slides using Mowiol and viewed by direct immunofluorescence using the Leica inverted confocal microscope (DM IRE2). Image J was used to analyse the images. Each experiment was repeated at least three times. 50 cells were counted for quantification purposes.

### Mass Spectrometric Identification of Sites of Phosphorylation

ROR2-myc was co-expressed with active Src (Src A) or inactive Src (Src MF) for 24 h in T/C 28a2 chondrocytes. ROR2-myc was immunoprecipitated using anti-myc antibody and was separated by SDS-PAGE. Following Coomassie staining, the ROR2-myc band was excised. Cysteines were reduced (10 mM dithiothreitol) and alkylated (50 mM iodoacetamide) prior to overnight in-gel trypsin digestion (12.5 ng/µl; Trypsin Gold; Promega, Madison, WI, USA) in 25 mM ammonium bicarbonate.

Phosphopeptides were enriched from the resulting mixture by TiO_2_ affinity chromatography according to Larsen *et al.* (25), with minor modifications. Peptides were loaded onto TiO_2_ micro-columns in 2% TFA. Columns were washed with 100 mg/ml DHB, 80% MeCN, 2% TFA, then with the same buffer omitting DHB. Peptides were eluted in a two-step procedure with 50 mM Na_2_HPO_4_ followed by dilute NH_4_OH solution. Eluates were desalted using ZipTips (Millipore). The resulting peptide mixtures (eluates and un-bound fraction) were analysed by liquid chromatography mass spectrometry (LC-MS/MS).

On-line liquid chromatography was performed by use of a Micro AS autosampler and Surveyor MS pump (Thermo Electron, Bremen, Germany). Peptides were loaded onto a 75 µm (internal diameter) Integrafrit (New Objective, USA) C8 resolving column (length 10 cm) and separated over a 40 minute gradient from 0% to 40% acetonitrile (Baker, Holland). Peptides eluted directly (∼350 nL/min) via a Triversa nanospray source (Advion Biosciences, NY, USA) into a 7 Tesla LTQ FT mass spectrometer (Thermo Electron). The mass spectrometer alternated between a full FT-MS scan (m/z 395-1600), subsequent CID MS/MS scans of the five most abundant ions, and, if a neutral loss of 98 Da from the precursor ion was observed in the CID mass spectrum, an MS^3^ scan of the neutral loss ion. Survey scans were acquired in the ICR cell with a resolution of 100,000 at m/z 400. Precursor ions were isolated and subjected to CID in the linear ion trap. Isolation width was 3 Th. Only multiply-charged precursor ions were selected MS/MS. CID was performed with helium gas at normalized collision energy of 35%. Precursor ions were activated for 30 ms. Data acquisition was controlled by Xcalibur 2.0 software. Data were searched against the Swissprot database using the SEQUEST (Thermo Electron) and Mascot algorithms (Matrix Sciences, UK). Phosphorylation site localisation was assessed using the A-score algorithm (26), with manual validation.

## Supporting Information

Figure S1 The annotated mass spectra of phosphorylation sites in the mouse ROR2 cytoplasmic regions. Eight Src-dependent phosphopeptides were identified by mass spectrometry.
(0.50 MB TIF)

Table S1 (0.07 MB DOC)

Table S2 (0.06 MB DOC)
