# Supplementary figures and images for: The Deleted in Brachydactyly B Domain of ROR2 Is Required for Receptor Activation by Recruitment of Src
Source: PLoS One. 2008 Mar 26;3(3):e1873. doi: 10.1371/journal.pone.0001873 (PMC2268744; doi:10.1371/journal.pone.0001873)

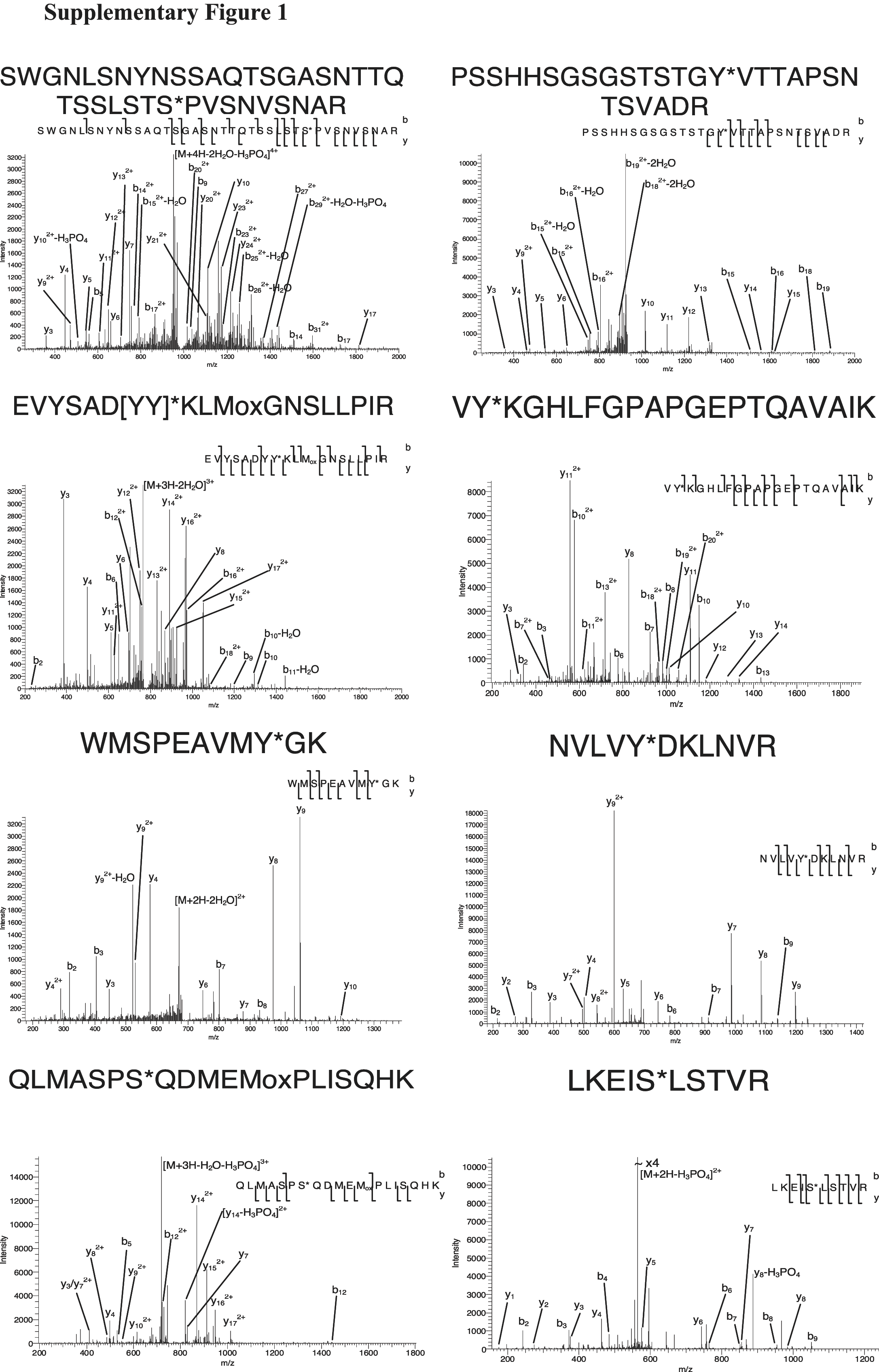

Supplement: Figure S1 — The annotated mass spectra of phosphorylation sites in the mouse ROR2 cytoplasmic regions. Eight Src-dependent phosphopeptides were identified by mass spectrometry. (0.50 MB TIF) [file pone.0001873.s001.tif]
