# Supplementary material for: The Deleted in Brachydactyly B Domain of ROR2 Is Required for Receptor Activation by Recruitment of Src
Source: PLoS One. 2008 Mar 26;3(3):e1873. doi: 10.1371/journal.pone.0001873 (PMC2268744; doi:10.1371/journal.pone.0001873)
Supplement: Table S1 — (0.07 MB DOC) [file pone.0001873.s002.doc]

**Supplementary Table 1-** The mouse ROR2 sequence coverage by mass spectrometry. 48.6% of amino acids were identified (Bold and underlined in red), including 9 out of 20 tyrosine residues in the cytoplasmic region. Peptides filtered using Bioworks 3.3 peptide probability: <1e-3.

| MARGWVRPSR | VPLCARAVWT | AAALLLWTPW | TAGEVEDSEA | IDTLGQPDGP | DSPLPTLKGY |
| --- | --- | --- | --- | --- | --- |
| FLNFLEPVNN | ITIVQGQTAI | LHCK**VAGNPP** | **PNVRWLKNDA** | **PVVQEPR**RVV | IRKTEYGSRL |
| R**IQDLDTTDT** | **GYYQCVATNG** | **LKTITATGVL** | **YVR**LGPTHSP | NHNFQDDDQE | DGFCQPYRGI |
| ACARFIGNR**T** | **IYVDSLQMQG** | TMIGTSTQLS | DQCSQFAIPS | FCHFVFPLCD | ARSRAPKPRE |
| LCRDECEVLE | NDLCR**QEYTI** | **ARSNPLILMR** | LQLPK**CEALP** | **MPESPDAANC** | GRYHQCYNGS |
| GADYRGMAST | TK**SGHQCQPW** | **ALQHPHSHRL** | **SSTEFPELGG** | **GHAYCRNPGG** | **QMEGPWCFTQ** |
| **NK**NVR**VELCD** | **VPPCSPR**DGS | SIAIPLVIAC | LFFLVCMCRN | KQKASASTPQ | RR**QLMASPSQ** |
| **DMEMPLISQH** | **K**QAK**LKEISL** | **STVRFMEELG** | **EDRFGKVYKG** | **HLFGPAPGEP** | **DKAEGPLREE** |
| **FRQEAMLR**AR | **LQHPNIVCLL** | **GVVTKDQPLS** | **MIFSYCSHGD** | **LHEFLVMRSP** | **HSDVGSTDDD** |
| **RTVKSALEPP** | **DFVHVVAQIA** | **VVHK**DLATR**N** | **VLVYDKLNVR** | **ISDLGLFREV** | **YSADYYKLMG** |
| **NSLLPIRWMS** | **PEAVMYGK**FS | IDSDIWSYGV | VLWEVFSYGL | QPYCGYSNQD | LPCPDDCPAW |
| VYALMIECWN | EFPSRRPR**FK** | **DIHSR**LR**SWG** | **NLSNYNSSAQ** | **TSGASNTTQT** | **SSLSTSPVSN** |
| **VSNAR**YMAPK | QK**AQPFPQPQ** | LVPPAQLYIP | VNGYQPVPAY | GAYLPNFYPV | QIPMQMAPQQ |
| VPPQMVPK**PS** | **SHHSGSGSTS** | **TGYVTTAPSN** | **TSVADR**AALL | SEGTEDVQNI | EAEEEEEGSV |
| PETELLGDND | TLQVTEAAHV | QLEALEQKLI | SEEDL |  |  |
