# Supplementary material for: The Deleted in Brachydactyly B Domain of ROR2 Is Required for Receptor Activation by Recruitment of Src
Source: PLoS One. 2008 Mar 26;3(3):e1873. doi: 10.1371/journal.pone.0001873 (PMC2268744; doi:10.1371/journal.pone.0001873)
Supplement: Table S2 — (0.06 MB DOC) [file pone.0001873.s003.doc]

**Supplementary Table 2**- The phosphorylation sites in the mouse ROR2 cytoplasmic regions. The identified sites of phosphorylation included one tyrosine within the C-terminal domain and four tyrosines within the kinase domain.

| Mass (Da) | Phosphopeptide | Site | Mass accuracy (ppm)^1^ | z^2^ | Mascot Score^3^ | Localisation (**A Score**)^4^ | Src dependent?^5^ |
| --- | --- | --- | --- | --- | --- | --- | --- |
| 2265.98 | QLMASPS*QDMEMoxPLISQHK | Ser 449 | 5.5 | 3 | 60 | 31 | Yes |
| 1224.65 | LKEIS*LSTVR | Ser 469 | 1.8 | 2 | 50 | 33 | Yes |
| 2259.15 | VY*KGHLFGPAPGEPTQAVAIK | Tyr 488 | 3.3 | 3 | 63 | 240 | Yes |
| 1411.72 | NVLVY*DKLNVR | Tyr 624 | 1.2 | 2 | 58 | NA | Yes |
| 2327.09 | EVYSAD[YY]*KLMoxGNSLLPIR | Tyr 645/646 | 3.9 | 3 | 38 | *15; Tyr 646* | Yes |
| 1377.55 | WMSPEAVMY*GK | Tyr 666 | 3.8 | 2 | 38 | 106 | Yes |
| 3941.73 | SWGNLSNYNSSAQTSGASNTTQTSSLSTS*PVSNVSNAR | Ser 776 | 3.8 | 4 | 62 | 21 | Yes |
| 2827.20 | PSSHHSGSGSTSTGY*VTTAPSNTSVADR | Tyr 873 | 0.2 | 3 | 58 | 31 | Yes% |

^1^ Mass accuracy obtained with external calibration

^2^ z: charge-state of peptide

^3^ Mascot scores >31 are significant

^4^ **A score** >20 indicated unambiguous localisation (ref 1)

^5^ Phosphopeptides were observed with co-transfection of activated Src, not with kinase-dead Src

% Phosphopeptide present in kinase-dead Src too, but 10-100x lower intensity and appears not to be pTyr

Kinase domain: 465-749 (blast homology)
